# Calcinosis universalis in juvenile dermatomyositis

**DOI:** 10.11604/pamj.2024.48.132.38591

**Published:** 2024-07-24

**Authors:** Maryem Ferjani, Mounira El Euch

**Affiliations:** 1 Pediatrics Department Charles Nicolle Hospital of Tunis, Tunis, Tunisia,; 2 University of Tunis El Manar, Tunis, Tunisie,; 3 Internal Medicine Department “A”, Charles Nicolle Hospital, Tunis, Tunisia,; 4 Research Laboratory of Kidney Diseases, Charles Nicolle Hospital of Tunis, Tunis, Tunisia

**Keywords:** Juvenile dermatomyositis, calcinosis, pediatrics

## Image in medicine

A 5 years-old female Tunisian child was admitted in our department for inflammatory polyarthralgia, progressive limb weakness, severe muscle fatigability and fever. On physical examination she had heliotrope eyelid edema, extensive calcalreous depositis in her four extremities, trunk, abdomen and on the back with Gottron' papules. Muscle enzymes were high particularly Creatinine Phosphokinase (CPK). Electromyography showed a myogenic process and muscle biopsy revealed characteristic signs of inflammatory muscle disease. Radiographic studies revealed extensive deep muscular calcareous deposits regarding joints. Juvenile Dermatomyositis (DM) complicated with calcinosis universalis was retained. Although intensive glucocorticoids combined with methotrexate was given, the calcific nodules gradually increased in size, ulcerated frequently and restricted her joint mobility. This case illustrates an extreme rare form of calcinosis which occurs more commonly in juvenile DM than in adult DM. There is until now no successful treatment of calcinosis universalis identified to date. Few reports described successful treatment with immunoglobulins, probenecid, aluminum hydroxide, warfarin, and diltiazem. Further investigation of disease mechanisms is needed so more effective therapeutic strategies may be developed.

**Figure 1:**
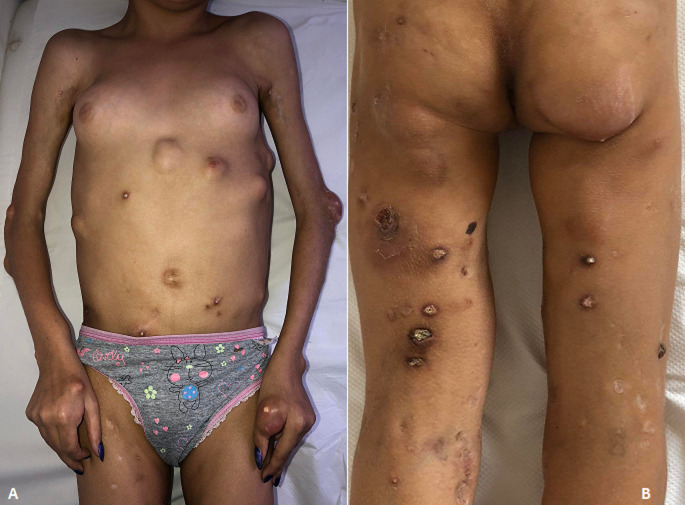
A) extensive calcareous deposits and multiple nodules in upper extremities and joints; B) calcinosis and nodules on the back

